# Compulsive Internet and Prevalence Substance Use among Spanish Adolescents

**DOI:** 10.3390/ijerph17238747

**Published:** 2020-11-25

**Authors:** Sonia Fernández-Aliseda, Angel Belzunegui-Eraso, Inma Pastor-Gosálbez, Francesc Valls-Fonayet

**Affiliations:** 1 History Department (Sociology Section), Universitat Rovira i Virgili, 43002 Tarragona, Spain; sonia.fernandeza@urv.cat (S.F.-A.); inma.pastor@urv.cat (I.P.-G.); 2 Medical Anthropology Research Centre, Universitat Rovira i Virgili, 43002 Tarragona, Spain; 3 Faculty of Nursing, Universitat Rovira i Virgili, 43002 Tarragona, Spain; francesc.valls@urv.cat

**Keywords:** compulsive use of the internet, teenagers, substance use, addiction, alcohol, tobacco

## Abstract

This paper analyses compulsive Internet use among Spanish adolescents as measured by the Compulsive Internet Use Scale (CIUS) of the ESTUDES 2016 survey (national survey on drug use in secondary schools), which was recently added to the statistical programme of the Spanish National Plan on Drugs. We examined two subsamples of Spanish adolescents (those who suffer from compulsive Internet use and those who do not) while taking into account gender and age. Our general hypothesis was that adolescents who suffer from compulsive Internet use have a greater prevalence of alcohol, tobacco, cannabis, sedative, and new substance consumption as well as a greater prevalence of modes of consumption such as getting drunk, drinking with friends in public places (*botellón*), and binge drinking. While our results confirm these assumptions, they also suggest that gender and age play an ambivalent role in these associations.

## 1. Introduction

The younger generations have grown up in an era when the Internet has become a central feature of their lives [1]. The habitual use of Information and Communication Technologies (ICT) is especially consolidated in the young and adolescent populations [2], and the use of such technologies is especially widespread among 17 and 18 year olds [3,4]. Personal relationships, social interactions, studies, leisure activities, and all kinds of information searches are mediated by the Internet and the social networks [5]. We have moved from the so-called Gutenberg Galaxy to what we might call the Internet Galaxy, i.e., the global-relational context in which the new generations are conducting most of their activities.

At the transitional stage of adolescence, Internet consumption patterns can develop that may lead to addiction, or at least to problematic uses [6,7,8] that can have negative consequences on a teenager’s psychosocial well-being [9]. At the same time, it is at this stage of life that the consumption of the most prevalent substances (alcohol, tobacco and, to a lesser extent, cannabis and others) begins. Alcohol-related socialization behaviors, such as drinking at parties and in outdoor public spaces, as well as forms of consumption such as binge drinking, also appear at this age. However, few studies have analyzed these phenomena jointly (i.e., on one hand, the degrees of Internet consumption and, on the other, the consumption of substances such as alcohol, tobacco, cannabis, and the practices in which this consumption occurs). The study by Sánchez-Martínez and Otero-Puime [10], conducted with high school students in Madrid, gives an indication of the association between Internet addiction and health risk behaviors such as alcohol and tobacco consumption. One of their conclusions, for example, shows that adolescents who have engaged in excessive consumption of alcohol are at greater risk of high Internet use. Another of their findings was an association between very high Internet use and depression. In a sample of university students, Fernández-Villa et al. [11] also found an association between the greater use of Internet on the one hand and greater levels of alcohol consumption, sleep disturbance, or low self-esteem on the other.

Our analysis aims to shed light on the phenomenon of Internet use and determine whether the profile of those who present compulsive use also present a greater prevalence of substance use. This type of analysis is still rare since Internet addiction and the consumption of substances such as alcohol, tobacco, and cannabis are normally treated separately. Increasingly, the complex phenomenon of addictions at stages of life such as adolescence requires the analysis of possible connections between the various consumption behaviors, especially those that carry the risk of addiction. Since the consumption of Internet content and the onset of substance use coincide in adolescence, the extent to which these phenomena may be linked requires examination. This analysis is important because it provides information that will help to design better prevention programmes for the adolescent population. It can also be used to inform health and education professionals about the profiles of consumers and enable them to detect risky behaviors.

However, the way Internet consumption should be measured has not reached general consensus in Spain, where different instruments have been used. For this study, our novel approach is to use the Compulsive Internet Use Scale (CIUS) scale, which was recently added to the biannual statistical programme of the National Plan for Drugs introduced by the Spanish Ministry of Health.

In this study we used the concept of compulsive internet use proposed by Young [12] to define aspects such as recurring thoughts about the Internet, the need to increase connection time, and the inability to control connection time. The literature has also highlighted certain symptoms associated with compulsive use, including anxiety and depression [13], changes to daily lifestyle activities, such as less contact with family and friends, and impact on hygienic and health habits (e.g., less physical activity, a greater prevalence of eating disorders, and changes to the sleep–wake cycle) [14].

The Compulsive Internet Use Scale (CIUS) is based on DSM-IV criteria for substance addiction and pathological gambling. With a maladaptive preoccupation with Internet use, as indicated by at least one of the following: (A) preoccupation with Internet use that is experienced as irresistible; (B) excessive use of the Internet for longer periods of time than planned. 2. Internet use, or preoccupation with its use, causes clinically significant distress or impairment in social, occupational, or other important areas of functioning. 3. Excessive Internet use does not occur exclusively during periods of hypomania or mania and is not better accounted for by other Axis I disorders [15]. The scale comprises 14 Likert-scale items and has a theoretical value range of 0–56 (see the Methodology section for an explanation of the items and measurement range for this scale). The items cover five symptoms of addiction: loss of control, withdrawal, mood modifications, preoccupations and conflicts [16]. A score ≥28 indicates compulsive Internet use.

This article therefore aims to provide evidence on compulsive Internet use (CU) among Spanish adolescents as measured by the national survey on drug use in secondary schools (ESTUDES) of the National Plan on Drugs promoted by the Spanish Ministry of Health, Consumption and Social Welfare. In the next section we analyze several studies that have tackled this subject from different approaches. In the Methodology section, we present our sample, discuss the instruments for this analysis, and describe several properties of the CIUS scale, which forms part of the ESTUDES questionnaire. We then present our most relevant results before opening our discussion and stating the conclusions we have drawn from our analysis.

## 2. A Review of Studies on Internet Use

The immediacy of telematic connections today is changing relational patterns especially among young people in our technologically advanced societies. Devices such as smartphones, tablets, and laptops are inseparable components of most activities children and adolescents conduct in their daily lives. These devices are essential to their relationship patterns because they enable them to create and strengthen interpersonal relationships, support others, receive social support, cultivate emotional bonds [17,18,19,20], and stay permanently connected with their friends [21].

However, intensive use of these tools can sometimes have negative consequences for young people. These include isolation and loneliness [22], connectivity stress, cyberbullying and cybercontrol [23], lack of privacy caused by overexposure, addiction, etc., all of which are considered by the World Health Organization to be new public health problems. Whatever the case, teenagers’ use of the Internet is causing concern [24].

Some authors believe that adolescents’ excessive use of social networks begins when they spend more than two hours a day on the Internet [25]. Others assert that Internet use becomes even more problematic when teenagers are connected for long periods at night. It may even be considered a form of behavioral addiction if teenagers spend excessive lengths of time connected, stop doing other activities in order to remain connected, or wish to be connected when they are unable to, etc. [26,27,28]. Such behaviors can lead to problems in teenagers’ daily lives, including poor academic performance and absenteeism, withdrawal from genuine social activities [29], alterations to their eating and sleeping patterns, as well as other problems that are highlighted in the literature [30].

Studies in the literature reveal a discrepancy regarding whether gender is a variable associated with problematic Internet use [31]. While some authors assert that there are no important differences between the genders and that connectivity technologies are used as a socialization tool equally by both sexes [32,33], others have found a higher prevalence of problematic Internet use in males [34,35] while still others have found that the prevalence is higher in women [36]. A study conducted in six European countries concluded that women are more likely to present symptoms compatible with an addiction such as the excessive use of social networks [37] and to experience unease or anxiety when they do not have access to a mobile phone [38].

Generally speaking, the literature suggests a differential use of the Internet and social networks between male and female adolescents. Teenage girls tend to use the Internet more to find information about their studies or employment [39,40] and to access instant messaging applications [41], email [42,43], or social networks. Teenage boys, on the other hand, use it more for video games and online gaming applications [44], downloads [45], and accessing websites with pornographic content [46].

In the case of Spanish adolescents, the ESTUDES survey shows that total compulsive Internet use was 16.4% in 2014 and 23% in 2016 of the students. If we look at gender differences, the figures were 14.7% (boys) and 18% (girls) in 2014 and 20.3% (boys) and 26.1% (girls) in 2016. These data provide a strong argument for asserting that compulsive Internet use is suffered by both sexes, which is in agreement with studies that found similar patterns [47].

The compulsive and/or problematic use of the Internet by adolescents can lead to verbal and nonverbal disconnection between family members, poorer relationships with parents [48], misunderstandings and distancing [49], especially in families with a sizeable generational difference between parents and children [50], and the search for social support on the Internet in cases where children and adolescents lack parental support [51].

Moreover, some studies suggest that the extent of parental control affects a teenager’s likelihood of facing risks on the Internet [52]. Direct parental rules that limit an adolescent’s use of applications or restrict their activities are associated with a less excessive Internet use by those adolescents [53]. However, restrictive or excessive parental control can also become a source of family conflict [54] and lead to lack of trust and poorer communication on the part of the adolescents involved [55].

Parental control depends on numerous variables, including age and gender. For example, parental control of Internet use tends to decrease as children grow older, which is consistent with the progressive need of teenagers for greater autonomy and independence as they transition towards adult life [56,57]. With regard to gender, however, the results are less consistent. Some studies have found no statistically significant differences between boys and girls with regard to parental control [58] while others assert that girls tend to be subjected to tighter control and supervision by their parents [59].

In line with the aim of this article, several studies have associated an excessive use of the Internet with a greater consumption of alcohol, tobacco, and other substances. For example, it has been observed that problematic Internet users may generally be up to three times more likely to develop risky use of alcohol and almost four times more likely to develop risky drug use [60].

Logistic regression analysis has also revealed a greater prevalence of risky alcohol consumption among adolescents who engage in problematic Internet use and shown that this prevalence is greater among teenage girls than among teenage boys [47].

Finally, though this point is not the purpose of this article, some of the issues that most concern researchers are practices such as cyberbullying, sexting, and grooming. The main studies conducted nationally and internationally on the issue of cyberbullying all indicate a greater predisposition among girls to become victims and among boys to become aggressors [61,62]. On the other hand, there is a general lack of studies on the issue of sexting. This practice involves the dissemination or publication of sexual content (usually photographs or videos) created by the senders themselves (usually girls feeling under pressure to do so) [63] using their mobile phones or other technological devices.

## 3. Materials and Methods

### 3.1. Design and Hypothesis

We conducted an observational, descriptive, and cross-sectional study by examining the ESTUDES 2016 survey. This survey is part of a statistical programme of the Spanish National Drug Plan, which began in 1994, and involves a random sample of the population at every secondary school in Spain. The analyses presented below, which were conducted with microdata from that survey, focus on the possible associations between compulsive Internet use on the one hand and the prevalence of substance use and the main consumption patterns on the other. Data disaggregated by gender and age were analyzed. Our hypothesis was that compulsive Internet use may be associated with a greater consumption of substances among adolescents (alcohol, tobacco, cannabis, sedatives, and new substances: keta, spice, synthetic marijuana, meow meow, flakka, Superman, etc., which mimic the effect of illegal drugs such as cannabis, cocaine, and ecstasy, are available in herb, pill, powder, liquid, or incense form). We also expected to find a statistically significant relationship between compulsive Internet use on the one hand and the various modes of alcohol consumption (getting drunk, *botellón*—It is about drinking in the company of friends in squares, in outdoor parking lots, and in other public and/or private outdoor areas-, and binge drinking) and tobacco consumption (vaping) on the other.

### 3.2. Sample Size

The survey was conducted among pupils aged 14–18 who attend secondary schools. Two-stage cluster sampling was employed in which the units for the first stage were 863 randomly selected educational centers and those for the second stage were 1726 classrooms. The survey was administered to all pupils in those classrooms. The final sample comprised 35,370 pupils. The maximum sampling error for a 95.5% confidence level and p = q = 0.5 was 0.5% for these Spanish students aged 14–18 years. The large sample used in this study (>100,000 population units) and therefore the very small sampling error mean that our results may be extended to the entire population in this age group.

To adapt the proportionality of the sample to the universe, the sample was weighted by Spanish autonomous community, whether the institution was a state or private school, and the type of studies these schools offered. The distribution of respondents by gender is shown in Table 1:

### 3.3. Variables

In the context of the 2017–2024 National Strategy on Addictions, the Spanish National Plan on Drugs incorporated nonsubstance or behavioral addictions as a new field in all its lines of action, placing special emphasis on (face-to-face and online) gambling and addiction to new technologies. Since 2014, the ESTUDES survey has included a module to determine the extent of compulsive Internet use and gambling among the adolescent population. CIUS (the Compulsive Internet Use Scale) is a validated scale [64] for the early detection of possible risk cases due to Internet use. It comprises a single dimension with 14 items measured by a Likert scale with the following five frequency categories: 0 = Never; 1 = Rarely; 2 = Sometimes; 3 = Often; 4 = Frequently. A score of at least 28 indicates risk of compulsive Internet use. The 14 items on the scale are as follows (Table 2):

Meerkerk et al. evaluated the one-factor structure and internal consistency of the CIUS [65]. The data for that study were gathered using an online survey conducted in 2002 with a sample of 1000 participants. The internal consistency in terms of Cronbach’s alpha was high (0.89).

Lopez-Fernandez et al. showed that the 14-item Compulsive Internet Use Scale (CIUS) is one of the most frequently internationally adapted psychometric instruments developed for assessing generalized problematic Internet use [66]. The data were collected via online surveys administered to 4226 voluntary participants from 15 countries, aged at least 18 years, and recruited from academic environments. The research groups in each country performed the translation (and back-translation) of the scale from the original language (English) into their respective languages (German, French, English, Finnish, Spanish, Italian, Polish, and Hungarian).

The above authors concluded that the CIUS is a reliable and structurally stable instrument that can be used for cross-cultural research across adult populations, especially if shortened versions of the scale are used [66].

An analysis by Sarmiento López et al. found that the scale’s adaptation and validation presented optimal values for supporting the consistency and quality of the construct. In addition, the fit to the original one-dimensional structure of the scale was also optimal [67]. When the scale was tested in three countries (Colombia, Mexico, and Spain), the internal consistencies for the three samples were 0.89, 0.92, and 0.95, respectively, with an average of 0.93 for the three samples as a whole.

With regard to sensitivity and specificity, some studies [65] show that the classification of cases is more consistent when the CIUS cut-off ≥ 18 (sensitivity 79.7%, specificity 79.4%). However, a higher cut-off (≥21) seems to be more suitable for estimating the prevalence of problematic Internet use [11] For Spain, Gómez Salgado et al. [68] concluded that this scale has sufficient theoretical and empirical support, including good psychometric properties (α = 0.83; specificity = 0.81; sensitivity = 0.80).

The consumption variables were dichotomous variables with Yes/No answers for the following types of consumption: Alcohol, Energy drinks mixed with alcohol, Tobacco (cigarettes), Cannabis, Sedatives and new substances (all related to consumption in the previous 30 days). Additionally, also used in relation to mode of consumption were Yes/No dichotomous variables (Getting drunk and Binge drinking in the previous 30 days, *Botellón* in the last 12 months, and Vaping at some time in my life).

### 3.4. Statistical Analysis

First, we calculated several psychometric properties of the CIUS scale, such as Cronbach’s alpha and the item-total correlations. We performed an analysis using Student’s *t*-tests for independent samples to determine whether there were any significant differences between teenage boys and teenage girls on each item and in the total scale. For comparisons of means and ANOVA, we calculated the effect sizes (Cohen’s d) in accordance with the instructions outlined by Rosenthal [69] and Rosenthal and DiMatteo [70].

We then calculated the odds ratio (OR) between the dichotomized variable Compulsive Use (CU)/Noncompulsive Use (NCU) and variables such as gender, prevalence of alcohol, tobacco, cannabis, sedative, and new substance consumption, and the getting drunk, *botellón*, and binge drinking modes of consumption. ORs are indicators of effect size, as are their confidence intervals, which, as Iraurgi [71] indicated, are used to determine the statistical significance observed in the analyses.

A 95% confidence level was considered for all analyses (*p* < 0.05). Data treatment was conducted using IBM-SPSS 21.0.

## 4. Results

Reliability analysis shows that the instrument demonstrates satisfactory reliability (Cronbach’s Alpha = 0.888) for the sample as a whole (N = 32,069). Table 3 shows the means for each item, the correlations between each item and the total scale, and Cronbach’s Alpha for the scale if a certain item is eliminated. As is shown by the latter statistic, the reliability of the scale does not improve if we delete the items one by one.

The mean of the CIUS scale for the sample as a whole is 19.87 points (Std Error = 0.058; 95% CI 19.76–19.99). The mean for teenage boys is 18.93 points (Std Error = 0.080; 95% CI 18.77–19.08) while that for teenage girls is 20.83 points (Std Error = 0.084; 95% CI 20.66–20.99). The *t*-test for the difference in means shows significant differences between the means of teenage boys and teenage girls, though the effect size is low (*t* = −16.37; *p* < 0.001; Cohen’s d = 0.183).

The *t*-tests also showed statistically significant differences for teenage boys and teenage girls in all items of the CIUS scale (Table 4):

Table 4 shows that although the *t*-tests indicate statistical significance for all items, the Cohen’s d coefficients for effect size suggest that some of these differences are of low strength [72].

From the items of the CIUS scale, we calculated the percentage of teenagers who may be considered within the compulsive use group (CU), which, as we mentioned earlier, are those who scored at least 28 points out of the total 56 points for the scale as a whole. We found that 23.2% (N = 7427) of adolescents are classified as belonging to the CU group, as opposed to 76.8% (N = 24,642) who scored less than 28 points. If we look at the figures by gender, we see that 26.1% of teenage girls are compulsive Internet users compared to 20.3% of teenage boys (Chi-Square = 153.258; *p* < 0.001; Cohen’s d = 0.1386). The odds ratio for gender and CU is OR = 1.39 (95% CI = 1.32–1.46), where male is taken as the reference variable. Neither the Chi-Square test nor the effect size or the OR show a sizeable association between CU and gender.

Age shows significant differences with respect to compulsive Internet use, but these differences are not linear. ANOVA analysis with CIUS dependent variables shows that teenagers aged 14 and 18 (the two extreme ages) (means = 19.29 and 19.33, respectively) score lower on the CIUS scale than those aged 15, 16, and 17, and that these differences are significant (F = 11.93; *p* < 0.001; Cohen’s d = 0.094), though the effect size is practically negligible. Adolescents aged 15, 16, and 17 do not present any differences (mean = 20.11, 20.27, and 20.02, respectively). This observation is maintained when the boys and girls of those ages are analyzed separately. However, when only adolescents who score at least 28 points are analyzed, i.e., those who display compulsive Internet use, the younger ones, i.e., the 14-year-olds, present slightly higher scores than the other teenagers (mean = 34.60) (see Table 5). There are no statistically significant differences among the subsample of teenagers who display compulsive Internet use (N = 7427) (F = 1.31; *p* = 0.265). Nor are there any gender-related differences in behavior in this subsample.

We then analyzed whether there were any differences between teenagers with CU and teenagers without CU in relation to their consumption of alcohol, cigarettes, vapers, cannabis, sedatives, and new substances. We also analyzed modes of consumption, in particular, in the case of alcohol, whether the teenagers engaged in activities such as getting drunk, *botellón*, and binge drinking. Table 6 shows the main results of these analyses.

When we control the compulsive Internet use group by gender, we observe the following significant associations in relation to the consumptions in Table 6: teenage girls with CU display higher percentages than teenage boys for getting drunk (31.1% compared to 25.9% for teenage boys with CU) and *botellón* (63.2% compared to 56.4%), whereas teenage boys with CU present higher percentages than teenage girls for binge drinking (26.8% compared to 24.3% for girls with CU) and vaping (27.5% compared to 22.9%). Teenage girls with CU had also consumed more sedatives in the previous 30 days (10.4%) than teenage boys with CU (7.0%). Teenage boys consume more energy drinks mixed with alcohol than teenage girls (22.6% compared to 18.7% for teenage girls). However, the prevalence of consumption of any alcoholic beverage in the previous 30 days was higher for teenage girls with CU than for teenage boys with CU (75.4% compared to 70.7%). Teenage girls with CU also consumed more cigarettes than teenage boys with CU (35.0% had smoked in the previous 30 days compared to 26.1% of teenage boys with CU). No significant differences were found in relation to the use of new substances between teenage boys and teenage girls with compulsive Internet use.

## 5. Discussion

The data we analyzed confirm the importance of considering problematic Internet use in certain addictive processes, as has been suggested by several studies that associate such use with certain psychopathological processes [73,74]. As we have seen, approximately 24% of adolescents aged between 14 and 18 use the Internet in what could be considered a problematic way (i.e., compulsive, according to the CIUS scale). When we controlled CU by age, we found that the intensity of CU among teenagers aged between 16 and 17 was the highest and that among teenagers aged between 14 and 18 it was the lowest. This result is consistent with the discrepancies found in the literature regarding whether Internet use normalizes with age [75] or whether problematic Internet use increases during adolescence [76].

Our analyses established an association between CU and the consumption of alcohol, cigarettes, cannabis, sedatives, and new substances in a manner that is consistent with other studies [77]. We also provide evidence that CU is also related to certain modalities of alcohol consumption, such as getting drunk, binge drinking and *botellón*, and to vaping.

However, our data suggest that caution should be exercised regarding the strength of the associations between CU and other variables, especially gender. This result is in agreement with those of other authors [78]. This indicates that caution should be exercised when analyzing the differences in means and confirms the results of earlier studies we mentioned that reported similar behaviors in relation to Internet use by teenage boys and teenage girls.

Although means comparison tests show differences for each CIUS scale item, Cohen’s d tests for effect sizes suggest that the sample size shifts the data towards certain associations that later appear to lack consistency, as Sun, Anderson, Palmer et al. also reported in their comparative study between American and Chinese adolescents [79]. According to our analyses, therefore, gender is not a clear determinant of the differences between CU and NCU since it is age rather than gender differences that has an effect. Interestingly, however, when the group of teenagers who display compulsive Internet use is taken in isolation, significant differences do appear between teenage boys and teenage girls in relation to both the modes of consumption and the substances consumed (except in the case of new substances and cannabis). For example, teenage girls who display CU have a greater prevalence for engaging in activities such as getting drunk and *botellón*, whereas teenage boys are more prone to binge drinking. In general, girls with CU were greater consumers of alcohol and cigarettes in the previous 30 days, while teenage boys with CU consumed more energy drinks mixed with alcohol.

CU seems increasingly to be becoming a commonplace in the lives of a large proportion of teenagers. That is, most teenagers who display compulsive Internet use appear not to be individuals who are excluded or self-absorbed but who carry out their daily activities with a greater or lesser degree of normality. For example, 51.1% of teenagers with CU play some sport or other between one and four times a week while 13.7% do so between five and seven times a week. Additionally, 11.3% read books between 1 and 3 days a week, while 7.7% do so between 5 and 7 days a week. Similarly, 52% regularly go out with their friends at least twice a week for walks, shopping or hanging out in the park, while 13.9% do so practically every day. These data are not very different from those of teenagers who do not display compulsive Internet use. However, teenagers with compulsive Internet use do perform more Internet downloads and view more videos and TV series, though no differences are observed in their use of social networks or instant communication networks.

## 6. Limitations

One limitation of this study is the use of the YES/NO dichotomous variable for the various types and forms of consumption, which reduces the amount of information collected and masks the intensity of consumption. For example, an adolescent who has consumed only one beer in the last 30 days is considered, for analytical purposes, in just the same way as an adolescent who has consumed one (or more) beers on each of those days. This is a big problem that should be solved in subsequent analyses that focus on intensity of consumption. Taking intensity into account could lead to changes in the effect sizes observed both among adolescents with compulsive Internet use and among those without, as well as between sexes and ages.

Another limitation is that these results do not enable a cause-and-effect relationship to be deduced between compulsive Internet use and substance use, though a profile for adolescents who combine these consumptions has been verified. Future research should determine how other intermediate variables act in this potential relationship. It would also be interesting to include a greater number of variables to evaluate along with the CIUS, since this would enable scholars to examine the relationship between compulsive use and other psychological variables such as sexting, gaming, and compulsive online shopping. In other words, adapting the CIUS to measure specific Internet activities would also contribute to the research process.

Another issue to consider is the general validity of the CIUS scale model. Authors such as Lopez-Fernandez et al. suggest using shorter versions of the scale since they have verified that such versions present greater reliability [74,75]. Further studies that apply CIUS in Spain should consider using shortened versions of CIUS, as has been suggested elsewhere.

Finally, this study did not take into account the influence of third variables that may be related to those we used in our regression analysis, e.g., disorders existing prior to Internet addiction and/or substance use, emotional distress, and parental control. This could lead to confounding bias. Later analyses should therefore incorporate intermediate variables that may modify the effect sizes.

## 7. Conclusions

These results provide evidence of the association between compulsive Internet use and the consumption of substances such as alcohol, tobacco, cannabis, sedatives, and new substances. Patterns of relationship between compulsive use and a greater prevalence of consumption have been identified. However, the associations found do not present large effect sizes, which indicates that adolescents who do not present compulsive Internet use also regularly consume these substances. In other words, the consumption of substances such as alcohol is a deep-rooted and widespread phenomenon among Spanish teenagers aged between 14 and 18 years old. The fact that few gender or age differences are observed also demonstrates that certain uses are socialized among Spanish adolescents. Our results have shown that girls display a greater compulsive Internet use than boys but that compulsive use does not present significant differences by age. This indicates that compulsive use, once it occurs, remains practically unchanged across this age range (14–18 years).

In a previous study we reported that teenagers who perceive themselves to be better informed also present a greater prevalence of consumption of practically all these substances [80]. This result, which we have called the information paradox, should help to focus the debate on how policies aimed at preventing consumption should be developed.

In summary, the data confirm our initial hypothesis that there is a statistically significant relationship between compulsive Internet use and the various modes of consumption analyzed. For all substances and modalities, there is a greater probability of consumption among adolescents who display compulsive use of the Internet. In our analysis we also included the so-called new substances. Although the prevalence of their consumption is low, questions are raised as to how this issue will evolve. Although we can affirm that the pattern of consumption is higher among adolescents with compulsive Internet use for every dependent variable, from the effect sizes expressed by the ORs we can also conclude that there are no great differences between teenagers who display CU and those who do not. The association between substance use and compulsive Internet use may be part of the same problem. It may also result from an association that is not one of causality but one that is mediated by other variables that do not appear in the analysis models. These variables include emotional distress, parent–children relationships, the feelings of uncertainty with which teenagers develop and which affect their family environments, and the fear of failure in a society that increasingly evaluates individuals for their achievements rather than for their efforts. Future analyses should therefore include such variables as predictors in models that aim to provide a more comprehensive analysis of this issue.

## Figures and Tables

**Table 1:** Number (percentage) of subjects in the sample by age and gender.

	14 Years Old	15 Years Old	16 Years Old	17 Years Old	18 Years Old	Total
Men	4535	3783	4660	3615	1288	17,881
	(12.8%)	(10.7%)	(13.2%)	(10.2%)	(3.6%)	(50.6%)
Women	4412	3555	4713	3685	1124	17,489
	(12.5%)	(10.1%)	(13.3%)	(10.4%)	(3.2%)	(49.4%)
Total	8947	7338	9373	7300	2412	35,370
	(25.3%)	(20.7%)	(26.5%)	(20.6%)	(6.8%)	(100%)

Source: authors’ own from ESTUDES 2016 data.

**Table 2:** CIUS scale items.

Item 1	How often have you found it difficult to stop using the Internet when you are online?
Item 2	How often have you continued to use the Internet despite your intention to stop?
Item 3	How often have your parents or friends told you to use the Internet less?
Item 4	How often have you preferred to use the Internet rather than spending time with others (e.g., your parents or friends)?
Item 5	How often are you short of sleep because of the Internet?
Item 6	How often do you think about the Internet even when you are not online?
Item 7	How often do you look forward to your next Internet session?
Item 8	How often do you think you should use the Internet less often?
Item 9	How often have you tried unsuccessfully to spend less time on the Internet?
Item 10	How often have you rushed through your homework to get on the Internet?
Item 11	How often do you neglect your obligations because you prefer to go on the Internet?
Item 12	How often do you go on the Internet when you are feeling down?
Item 13	How often do you use the Internet to forget your sorrows or negative feelings?
Item 14	How often do you feel restless, frustrated, or irritated when you cannot use the Internet?

Source: authors’ own.

**Table 3:** Item-total statistics.

	Scale Mean if Item Deleted	Scale Variance if Item Deleted	Corrected Item-Total Correlation	Squared Multiple Correlation	Cronbach’s Alpha if Item Deleted
Item 1	18.09	94.76	0.607	0.446	0.879
Item 2	18.42	92.67	0.62	0.478	0.878
Item 3	18.18	93.71	0.555	0.356	0.882
Item 4	18.77	97.89	0.489	0.264	0.884
Item 5	18.56	94.96	0.53	0.304	0.883
Item 6	18.86	95.88	0.595	0.431	0.88
Item 7	18.25	94.30	0.646	0.481	0.878
Item 8	18.23	96.78	0.486	0.37	0.884
Item 9	18.79	95.88	0.556	0.414	0.881
Item 10	18.56	93.88	0.605	0.415	0.879
Item 11	18.68	94.96	0.577	0.386	0.88
Item 12	17.95	93.53	0.542	0.596	0.882
Item 13	18.13	92.89	0.536	0.595	0.883
Item 14	18.85	94.61	0.614	0.399	0.879

Source: authors’ own.

**Table 4:** *t*-tests for comparing the means of the teenage boys and teenage girls.

	Mean (Boys)	Mean (Girls)	*t*	Sig. (2-Tailed)	Cohen’s d
Item 1	1.66	1.91	−21.507	0.000	0.228
Item 2	1.33	1.58	−19.904	0.000	0.219
Item 3	1.61	1.77	−11.382	0.000	0.126
Item 4	1.12	1.08	4.22	0.000	−0.048
Item 5	1.28	1.35	−4.479	0.000	0.041
Item 6	1.05	0.97	7.412	0.000	−0.077
Item 7	1.59	1.67	−7.436	0.000	0.074
Item 8	1.50	1.78	−23.503	0.000	0.256
Item 9	0.98	1.18	−16.94	0.000	0.183
Item 10	1.37	1.26	8.678	0.000	−0.094
Item 11	1.22	1.15	5.924	0.000	−0.062
Item 12	1.72	2.11	−28.18	0.000	0.301
Item 13	1.54	1.94	−27.233	0.000	0.287
Item 14	0.96	1.09	−10.916	0.000	0.118

Note: the confidence level for all tests is 95%. Source: authors’ own from data extracted from the ESTUDES 2016 survey.

**Table 5:** Means on the CIUS scale by age.

Age	Total Sample (N = 32,069)	Sample: Compulsive Use (7427)
14	19.3	34.6
15	20.1	34.4
16	20.3	34.3
17	20.0	34.2
18	19.3	34.2

**Table 6:** Consumption by adolescents who display compulsive Internet use (CU) and those who do not (NCU).

	CU	NCU	OR	CI95%	Transformed OR (*OR_t_*) (1)
Alcohol (*)	70.4%	57.9%	1.72	[1.62–1.83]	63.3%
Energy drinks + alcohol (*)	20.5%	13.0%	1.72	[1.60–1.83]	63.2%
Getting drunk (*)	28.8%	19.7%	1.65	[1.55–1.75]	62.3%
Binge drinking (*)	25.4%	18.0%	1.55	[1.46–1.65]	60.8%
*Botellón* (**)	60.2%	49.8%	1.52	[1.44–1.60]	60.3%
Cigarettes (*)	31.1%	21.5%	1.65	[1.55–1.74]	62.2%
Vaping (***)	24.9%	18.3%	1.48	[1.39–1.58]	59.7%
Cannabis (*)	16.5%	13.0%	1.33	[1.23–1.43]	57.0%
Sedatives (*)	8.9%	5.0%	1.87	[1.70–2.07]	65.2%
New substances (*)	2.5%	1.4%	1.88	[1.58–2.25]	65.4%

CU = compulsive use; NCU = noncompulsive use. (*) Consumption in the last 30 days; (**) Consumption in the last 12 months; (***) Consumption at some time in my life. Binge drinking = drinking 5 units of alcohol in less than 3 h. (1) The Transformed OR was calculated from the expression ORt = OR(1+OR). Source: authors’ own from data extracted from the ESTUDES 2016 survey.
